# Smart Railway Traffic Monitoring Using Fiber Bragg Grating Strain Gauges

**DOI:** 10.3390/s22093429

**Published:** 2022-04-30

**Authors:** Bastien Van Esbeen, Cyrille Finet, Robin Vandebrouck, Damien Kinet, Kevin Boelen, Corentin Guyot, Georges Kouroussis, Christophe Caucheteur

**Affiliations:** 1 Advanced Photonic Sensors Unit, Electromagnetism and Telecommunication Department, University of Mons, Boulevard Dolez 31, 7000 Mons, Belgium; bastien.vanesbeen@umons.ac.be (B.V.E.); cyrille.finet@student.umons.ac.be (C.F.); robin.vandebrouck@umons.ac.be (R.V.); damien.kinet@umons.ac.be (D.K.); 2 B-SENS, Boulevard Dolez 31, 7000 Mons, Belgium; kevin.boelen@b-sens.be (K.B.); corentin.guyot@b-sens.be (C.G.); 3 Theoretical Mechanics, Dynamics and Vibrations Department, University of Mons, Boulevard Dolez 31, 7000 Mons, Belgium; georges.kouroussis@umons.ac.be

**Keywords:** sensors, fiber bragg gratings, railway, safety

## Abstract

There is today ample evidence that fiber Bragg gratings (FBGs) distributed along a railway track can provide robust axle counting and bring numerous assets compared to competing technologies in this practical environment. This work brings two relevant originalities with respect to the state-of-the-art solutions. First, a study of the strain distribution in the rail cross-section is performed to determine the sensitivity according to the charge and the position on the rail. Secondly, the technology is deployed along the rail track as a smart object where the sensor head is composed of four FBG wavelength-division-multiplexed in a single telecommunication-grade optical fiber and interrogated by a miniaturized read-out device. Two FBGs ensure the detection of the train direction and another two bring the required redundancy to reach a safety integrity level (SIL) 4. The read-out unit has been specifically developed for the application and contains a vertical-cavity surface-emitting laser (VCSEL) and a photodiode driven by a high-speed microprocessor unit that processes the data and communicates the useful information, i.e., the number of axles. On-field tests confirm that the proposed approach makes the installation process easier while it democratizes the technology.

## 1. Introduction

Fiber Bragg gratings (FBGs) are a commercially-viable sensing technology that tends to outperform classical solutions in a growing number of industrial applications. Their accuracy and reliability have made them key components for e.g., temperature, strain and pressure sensing, in harsh environments or wherever electrical sensors pose a safety risk or when miniaturization is essential. Since the first report of their capability to effectively detect train axles [1], FBGs have been attracting growing attention in the traffic monitoring domain, which is extremely exigent in terms of safety. Any device developed to that aim should guarantee the highest safety integrity level (SIL), i.e., SIL 4 for which the probability of dangerous failure per hour should lie between 10^−8^ and 10^−9^ [2].

Railway safety is usually ensured through the use of specific devices (track circuits and axle counters) regularly placed along the rail tracks. A track circuit is a simple electrical device that can detect the presence of a train as well as a frank rail breakage. An axle counter operates between two counting heads installed at both ends of a rail section. Besides these classical metrological solutions, investigations are carried out to evaluate the potential of emerging technologies. Due to their intrinsic advantages (electromagnetic immunity, small size, low attenuation, robustness, real-time demodulation and remote interrogation of a several tens of cascaded sensors along a single fiber, among others), FBG sensors have been largely developed for railway monitoring. Today, there is no doubt that FBGs are effective axle counters when they are positioned along the track [1,3,4,5,6,7,8,9] and that they can bring additional features such as weigh-in-motion monitoring [10], wheel flat detection [11] and structural health monitoring (SHM) [12,13]. Despite these existing features and an undeniable rise in technology readiness level, there is still room for progress before the technology can be massively accepted for practical application in railway monitoring.

In this roadmap towards the production of industrial FBG strain sensors for railway safety applications, the work reported in this paper brings original and highly relevant practical studies. Considering the lack of unified information in the literature so far, an experimental study was first performed to determine the strain distribution in the rail section according to the charge. To this aim, a section of rail was placed in a mechanical press and nine FBGs were used for measurements at different positions of the web, the foot and the base of a flat-bottom rail. A cost-effective and miniaturized read-out device was then designed, able to interrogate four wavelength-division-multiplexed FBGs in a single-mode telecommunication-grade optical fiber. The four FBGs constitute an axle counting head. This number of four FBGs was selected according to our background in railway monitoring to enable the detection of the train direction (two cascaded gratings are required for that) and to reach SIL 4 operation (every sensor is doubled). Besides the redundancy, extensive field tests are also required to ensure SIL 4. The smart interrogator comprises a vertical-cavity surface-emitting laser (VCSEL) and a photodiode driven by a high-speed microprocessor. The latter processes the data in real time and communicates the useful information, i.e., the number of axles, so that the overall technology is now treated as a smart object. This makes the installation process easier, as there is no need to deploy the connecting optical fibers over large distances. Finally, on-field measurements on a railtrack section of the Belgian network were performed to validate all experiments conducted in the lab and determine the performance of the read-out technique. 

In the remainder of the paper, the FBG technology and the smart interrogator development are presented, followed by the experimental works conducted in the lab and on the field.

## 2. FBGs and Smart Interrogator

An FBG is a distributed mirror in a short segment of the optical fiber, reflecting a limited wavelength range around the so-called Bragg wavelength and transmitting all others [12,14,15]. The physical phenomenon behind this is a periodic and permanent modification of the core refractive index along the optical fiber axis induced by exposure of the core under ultraviolet light of an intense interference pattern. The index change is possible due to the fiber photosensitivity at a wavelength around 240 nm thanks to the presence of germanium oxide dopants inside the core. An FBG is defined by some physical parameters: the grating length L, the grating period Λ and the refractive index modulation *δn*. Optically, an FBG behaves as a narrow-band reflective filter around the Bragg wavelength:

(1) $$\lambda_{B}=2\left(n_{\mathrm{eff}}+\delta n\right)\Lambda \approx 2n_{\mathrm{eff}}\Lambda$$

where *n*_eff_ is the effective refractive index of the optical fiber. Any change of the effective refractive index or the grating period will induce a shift of the Bragg wavelength, which can be used to sense temperature, axial strain or pressure. Hence, the demodulation of an FBG sensor is based on tracking the shift of the Bragg wavelength as a function of the parameter to be measured. The FBG sensitivity to strain or temperature is linear and without hysteresis. Typical values of axial strain and temperature sensitivity at 1550 nm are 1.2 pm/µε and 10 pm/°C, respectively [5].

Uniform FBGs used in this work were produced in hydrogen-loaded single-mode telecommunication-grade optical fibers using the NORIA facility (Northlab Photonics, Nacka, Sweden). The latter encompasses a 193 nm excimer laser and phase masks to produce FBGs in a semi-automated way. Eight mm-long FBGs were produced in a couple of seconds with a repetition rate of the laser pulses set at 50 Hz. After the inscription, gratings were stabilized at 100 °C for 12 h to remove residual hydrogen and stabilize their physical properties. 

The vast majority of commercial FBG interrogators can be classified into two main groups: (1) highly-resolved but rather slow devices (repetition rate < 100 Hz, intrinsic wavelength resolution ~1 pm) based on a swept laser and a photodiode and (2) high-speed ones comprising a broadband source and a CCD (Charge Coupled Device) spectrometer (repetition rate > 1 kHz, wavelength resolution ~100 pm or even more). For this second family even more than for the first, peak-tracking algorithms are implemented to enhance the resolution. A good review of the main methods used to this aim can be found in [16].

**Figure 1:**
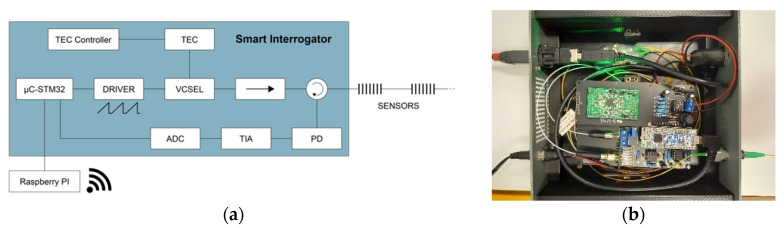
(**a**) Scheme of the developed smart interrogator; (**b**) picture of the developed printed circuit board for the smart interrogator.

To produce an FBG-based axle counter, a smart compact interrogator has been specifically developed, as sketched in Figure 1a. The light source is a VCSEL that brings numerous advantages, such as serial manufacturing, low threshold current, reduced footprint, low electrical power consumption and cost-effectiveness [14,17,18,19]. The VCSEL used in this study operates around 1550 nm—the most used wavelength range for telecommunications and fiber Bragg grating sensing—and can be tuned over a wavelength range of ~10 nm, allowing for reasonably interrogating a maximum of five FBGs cascaded along the fiber. The electrical driving unit has been made to modulate the electrical current in the VCSEL following a sawtooth function, consequently activating the VCSEL wavelength sweeping [17]. The VCSEL is temperature-stabilized using a thermoelectric cooler (TEC). An isolator is placed in front of the laser to avoid reinjection of light into the laser cavity, which can alter the laser. Light is launched into the FBGs through a circulator, to collect their reflected signal that is sent to an InGaAs PIN photodiode connected to a transimpedance amplifier (TIA). The output of the setup is a reflection spectrum composed of 200 data points obtained during the laser sweep period. It is then sent either to a computer or a Raspberry Pi 4 with a USB cable (serial protocol). The Raspberry Pi 4, located within the housing of the interrogator, is used for the sake of mobility where a remote user can visualize on his laptop (via a VNC connection) what is happening at the measurement points. The use of such a remote connection protocol tends to a smarter interrogator. Figure 1b displays a picture of the smart interrogator with its in-house developed printed circuit board.

**Figure 2:**
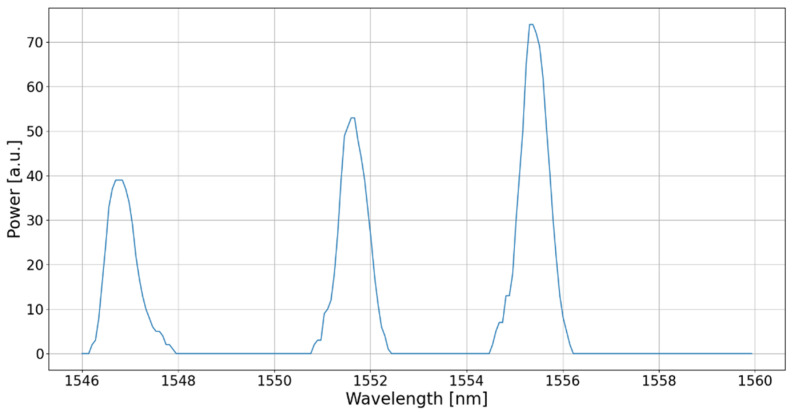
Reflected amplitude spectrum for three cascaded 1-cm long FBGs measured by the smart interrogator.

By means of a graphical user interface (GUI), the user is able to visualize the instantaneous reflected amplitude spectrum or the evolution of the Bragg wavelength of every FBG as a function of time. An example of a reflected amplitude spectrum measured by the interrogator in the case of a fiber composed of three FBGs is shown in Figure 2. In order to compute the instantaneous Bragg wavelength, a peak detection with the centroid method is implemented for a more accurate estimate of the Bragg wavelength, the peak detection alone being limited by the intrinsic device resolution (~47 pm). The centroid method consists of computing the center of mass of the data points that constitutes a reflection zone. This method was chosen due to its simplicity compared to other methods like a Gaussian curve fitting. Indeed, a Gaussian fit is an iterative method whose number of iterations depends on the initial conditions and can be about 200 times slower than the centroid method, which consists of a single computation. Hence, our choice allows for obtaining the highest possible repetition rate (>1 kHz). Let us note that, as in the example provided in Figure 2, the wavelength spacing between FBGs is chosen to avoid any issue due to possible drifts resulting from ambient temperature variations (50 °C or even more between summer and winter).

**Figure 3:**
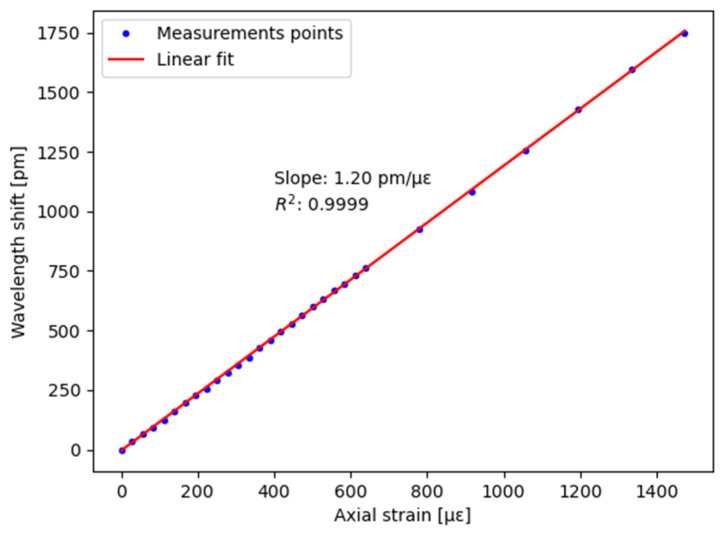
Calibration test of the smart interrogator for axial strain sensing.

To confirm the overall metrological performance of the interrogator, a calibration test was performed by applying calibrated axial strains on one FBG of the chain. A fiber section containing the grating was glued at both sides, and one end could be elongated by means of a micrometer positioning system. The test consisted of 24 strain states on a 72-cm long fiber section by steps of 20 µm (=27.8 µε) and six other strain states by steps of 100 µm (=138.9 µε). The averages of the measurement points of each strain iteration (which lasts a few seconds) are reported in Figure 3. The standard deviation is in the order of magnitude of 5 pm. A linear regression gives a slope of 1.20 pm/µε and a R^2^ of 0.999, which confirms the good operation of the smart interrogator. 

## 3. Laboratory Test

**Figure 4:**
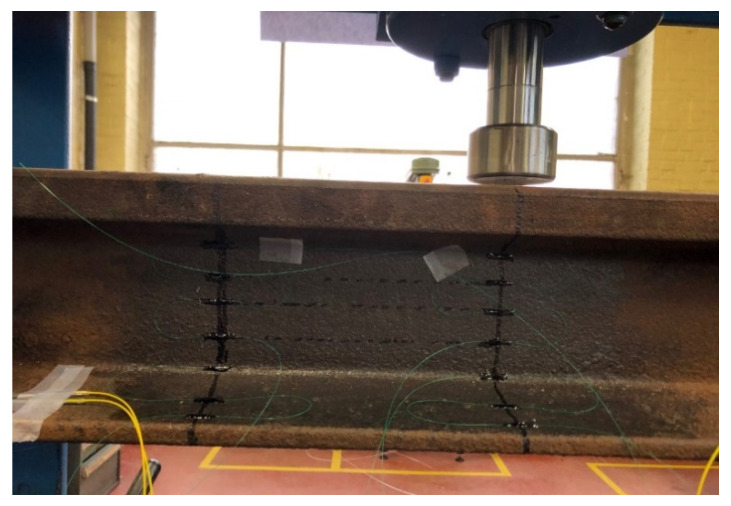
Three-point bending test of a rail (type 50E2) section for which nine FBG sensors are glued at different positions of the cross-section.

**Figure 5:**
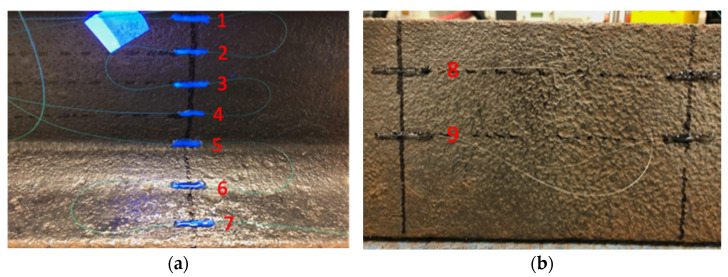
Pictures of the FBG placement: (**a**) On the web (1–5) and on the foot (6–7); (**b**) Under the foot (8–9).

In this section, experiments carried out to determine the optimal position of the FBGs on the rail cross-section are reported. To proceed, a 3-point bending was applied on a rail section using a mechanical press to simulate the weight induced by the wheel of a train, as shown in Figure 4. Nine bare FBGs were distributed on the rail. Sensors were glued using UV15DC80 (MasterBond, Hackensack, NJ, USA) adhesive. Figure 5 represents the placement of the FBGs: five on the web, two on the foot, and two under the foot. The bending test consisted of a gradual tonnage increase to provide the sensitivity (in pm/t) of each FBG. The sensors were connected to an FBG interrogator (other than the one of Section 2, able to interrogate up to twenty gratings in a fiber) to read-out their wavelength evolution and determine the sensitivity of the wavelength shift as a function of the load. The used device was the BSI-108 interrogator from B-SENS.

The obtained results are shown in Table 1. It appears that the most sensitive region of the rail is under its foot, with a sensitivity of ~32 pm/t, more than twice the sensitivity measured on the foot. One can also notice that a slightly negative sensitivity appears for sensor 1, which indicates the presence of the neutral axis between sensors 1 and 2, where the sensitivity remains null. 

**Figure 6:**
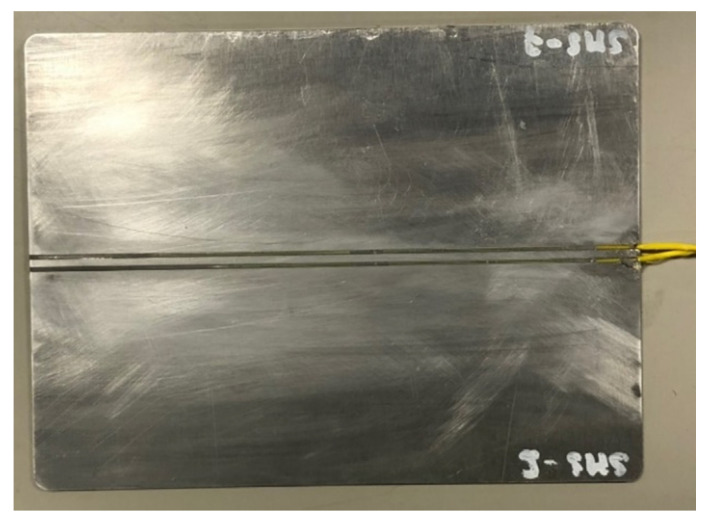
Picture of the aluminum PAD.

Based on these results, it has been decided to locate the sensors under the foot, as already done in previous works [5]. To enable a practical on-field installation, a specific packaging in the form of a pad has been developed. As the width of a rail foot (type 50E2) is 125 mm, the pad is 125 mm wide and 200 mm long. Two optical fibers (for redundancy), each containing a single FBG, are inserted in the middle of the pad. The latter is made in aluminum and contains two grooves in the center, allowing for positioning both optical fibers in parallel. The optical fibers protection is a polymer cover reinforced with Kevlar fibers. The packaging is shown in Figure 6.

Two additional 3-point bending tests were achieved with the pad glued under the foot of the rail. The sensitivities obtained from both tests are 24.2 pm/t. Even if the presence of the pad induces a decrease of the sensitivity of 25%, the resulting sensitivity is high enough to consider the packaging practically usable. Indeed, the weight of a 4-axle coach is about 50 tons, equivalent to roughly 6 tons per wheel; therefore, the Bragg wavelength shift will be over 100 pm.

## 4. On-Field Experiments

**Figure 7:**
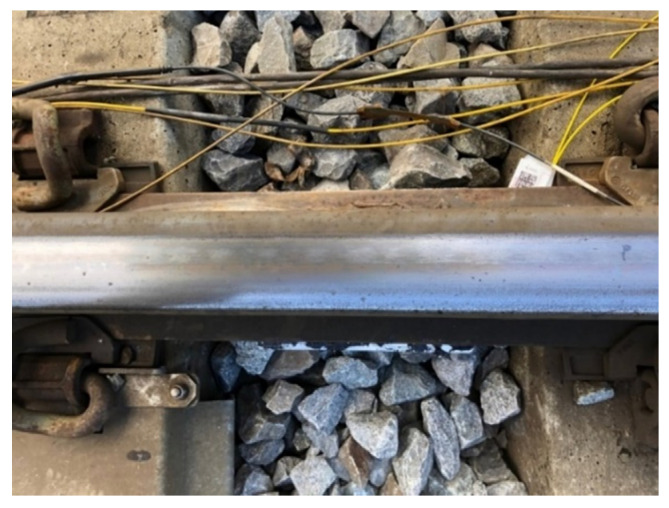
Picture of the setup: pad installed under the rail.

The pad containing the sensors was installed on a railtrack near the station of La Louvière-Sud in Belgium. To proceed with the installation, the ballast under the rail was first removed. Then, the rail foot had been polished to make the gluing process easier and a bi-component Protac 7300 adhesive was applied on the pad face. The pad was placed under the rail foot and kept in place for fifteen minutes by means of clamps specifically made for the occasion. After the clamps were removed, the pad properly stayed in place under the foot. The ballast was finally replaced. Figure 7 shows the top view of the rail with the packaging positioned between two consecutive sleepers.

**Figure 8:**
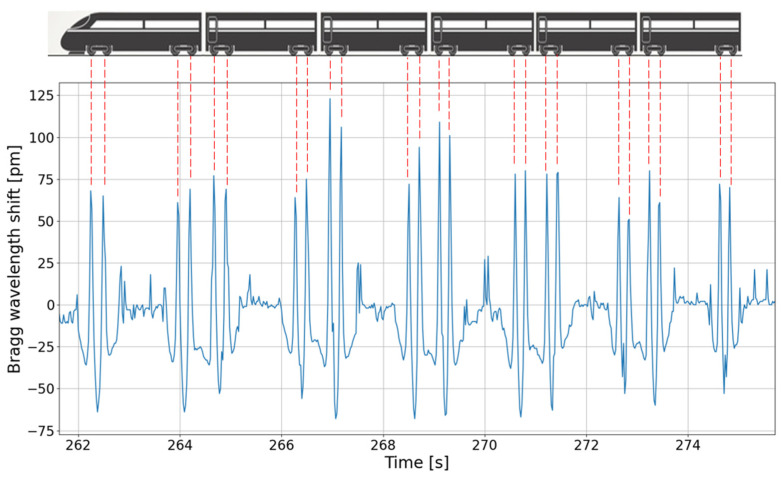
Evolution of the Bragg wavelength shift measured during the passage of a train.

Extensive train traffic measurements were done with the developed interrogator that was installed in an electrical cabinet, located a few tens of meters away from the measurement site. The device communicates its signal to the end user using an ethernet connection. Figure 8 shows an example of a trace (=Bragg wavelength evolution of a sensor as a function of time) for a train composed of six coaches. The peaks of the graph correspond to the instants at which the wheels of the trains are above the sensor. The installed setup thus operates as an effective axle counter.

## 5. Conclusions

In this paper, an axial strain calibration was first performed to demonstrate its good operation. In the lab using a mechanical press, an element of 50E2 rail was then subject to a 3-point bending test and a study of the sensitivity was achieved, as a function of the charge and the FBG position on the rail cross-section. The results show that the most sensitive position is under the rail, so an aluminum pad was designed to embed two fibers each containing two FBGs and to fit the dimensions under the foot of the rail. The on-field experiments confirm that a cost-effective smart interrogator, especially developed for this project, can be used for axle counting. Extensive tests are still required to demonstrate SIL4 operation.

## Figures and Tables

**Table 1:** Sensor sensitivity for each FBG on the rail.

FBG	L/2 [pm/t]
1	−1.1302
2	2.0697
3	5.7154
4	9.3414
5	14.1121
6	15.4327
7	14.7175
8	26.3303
9	32.0789

## Data Availability

Data underlying the results presented in this paper are not publicly available at this time but may be obtained from the authors upon reasonable request.
